# Strong genetic differentiation and low genetic diversity in a habitat‐forming fucoid seaweed (*Cystophora racemosa*) across 850 km of its range

**DOI:** 10.1111/jpy.70023

**Published:** 2025-05-03

**Authors:** Jane M. Edgeloe, Samuel Starko, Albert Pessarrodona, Melinda A. Coleman, Jacqueline Batley, Thomas Wernberg, Georgina V. Wood

**Affiliations:** ^1^ School of Biological Sciences University of Western Australia Perth Western Australia Australia; ^2^ Oceans Institute University of Western Australia Perth Western Australia Australia; ^3^ Department of Primary Industries National Marine Science Centre Coffs Harbour New South Wales Australia; ^4^ Norwegian Institute of Marine Research His Norway; ^5^ College of Science and Engineering, Flinders University Adelaide South Australia Australia

**Keywords:** DArTseq, genomics, macroalgae, natural selection, population structure, temperature, Western Australia

## Abstract

Temperate seaweed forests are among the most productive and widespread habitats in coastal waters. However, they are under threat from climate change and other anthropogenic stressors. To effectively conserve and manage these ecosystems under these rising pressures, an understanding of the genetic diversity and structure of habitat‐forming seaweeds will be necessary. Australia's Great Southern Reef, a global hotspot of endemic diversity, is home to one of the world's most speciose habitat‐forming seaweed genera, *Cystophora* (order Fucales). Despite severe declines in some species, genomic data on this genus remain limited. We used a reduced representation genomic approach (DaRTSeq) to investigate the genetic diversity and structure of *Cystophora racemosa*, a dominant canopy‐forming species, across ~850 km of its range. Our sequencing captured 4741 high‐quality single nucleotide polymorphisms (SNPs), and we distinguished neutral loci from those under natural selection (i.e., outlier loci). We identified strong population structure and high genetic differentiation for both neutral (mean *F*_ST_ = 0.404) and outlier loci (mean *F*_ST_ = 0.901). Across populations, genetic diversity was low (neutral: mean *H*_E_ = 0.046; outlier: *H*_E_ = 0.042), with high inferred inbreeding (neutral loci mean *F*_IS_ = 0.531) and no evidence of isolation‐by‐distance. Several SNPs (*n* = 70) were observed to be putatively adaptive, with most (97%) correlated with annual maximum sea surface temperature (SST, °C), indicating local adaptation to this key ocean variable. Our results show that *C. racemosa* populations have low genetic diversity and high differentiation, both of which may increase the vulnerability of this important foundation species to global change.

**Abbreviations**
AMOVA: analysis of molecular variance
AR: allelic richness
CE: cross‐entropy
DArT: Diversity Arrays Technology Pty Ltd.
*df*
: degree of freedom
FDR: false discovery rate
*F*_IS_
: inbreeding coefficient
*F*_ST_
: fixation index
GEA: Genotype‐Environment Association
GIF: genomic inflation factor
GSR: Great Southern Reef
*H*_E_
: expected heterozygosity
*H*_O_
: observed heterozygosity
HWE: Hardy–Weinberg equilibrium
IBD: isolation by distance
LFMM: latent factor mixed model
MAF: minor allele frequency
*N*_P_
: number of private alleles
PCoA: principal coordinates analysis
RDA: redundancy analysis
SNPs: single nucleotide polymorphisms
SST: sea surface temperature (°C)

## INTRODUCTION

Anthropogenic climate change is increasingly threatening global biodiversity, driving strong responses across marine ecosystems. Climate‐related stressors can evoke various consequences, including significant range contractions and local extinctions (see Pinsky et al., 2020; Smale & Wernberg, 2013; Starko et al., 2022), with numerous marine species either already experiencing declines or predicted to experience declines under future projected climatic scenarios (reviewed by Wesselmann et al., 2024). To persist through periods of climate change, species must either move (through geographic range shifts; Gervais et al., 2021; Parmesan, 1996; Parmesan & Yohe, 2003; Pinsky et al., 2020) or adapt, undergoing rapid evolution to increase their tolerance to increasing climate stress (Davis et al., 2005). Although the ability of species to adjust their ranges depends largely on dispersal ability (Alzate & Onstein, 2022; Lester et al., 2007), successful colonization and persistence in new environments are constrained by habitat availability, biotic interactions, and physiological tolerance (Berg et al., 2010). Moreover, the capacity of species to adapt depends strongly on the underlying genetic diversity of populations, which influences their potential for evolutionary responses to novel conditions (Frankham, 2005; Lande & Shannon, 1996; Lowe & Allendorf, 2010). Genetic diversity is a critical characteristic of populations that can bolster resilience against climate change (Davies et al., 2016; Frankel & Soule, 1981; Sgrò et al., 2011). Yet genetic diversity is also being eroded by many of the same stressors that organisms need to adapt to, such as temperature. Thus, understanding drivers of genetic diversity and genetic structure will aid in accurately predicting the impacts of climate change on species (Merilä & Hendry, 2014; Palumbi et al., 2019; Ramos et al., 2018; Scheffers et al., 2016).

One strategy for understanding the drivers of genetic diversity and structure across the range of a species is to separate genetic markers that show evidence of natural selection from those that appear to be structured by neutral processes such as genetic drift and historical processes (e.g., population bottlenecks; Kirk & Freeland, 2011). Although genetic variation at neutral loci does not directly influence fitness differences between populations, it provides valuable insights into gene flow, migration, and dispersal patterns within and among populations (Coleman et al., 2013; Dawson et al., 2005; Holderegger et al., 2006; Wilson et al., 2016). Studying neutral loci can provide insights into meta‐population dynamics, and advances in genomic approaches also allow us to identify genomic signatures of natural selection (reviewed in—Nielsen, 2005). Variation in allele frequencies at adaptive loci (hereafter adaptive genetic diversity) arises from natural selection (Fisher, 1930; Lewontin, 1974) and is directly associated with local adaptation to different environments (see Limborg et al., 2012; Schmidt et al., 2008; Vranken et al., 2021). Although putative adaptive loci may not be directly under selection, they may be physically linked to genomic regions that confer fitness advantages in specific environments, allowing insight into the degree to which natural selection is shaping genomic diversity (Corbett‐Detig et al., 2015; Gregory, 2009). Knowledge of both neutral and adaptive genomic information can help to assess extinction risks and develop management and conservation initiatives for threatened and declining species, specifically for endangered species with high intrinsic value (Domingues et al., 2018; Gose et al., 2024; Guzman et al., 2021; Lancaster et al., 2010) and foundation species such as seaweeds.

Large brown seaweeds (class Phaeophyceae) are among the most productive species globally (Pessarrodona et al., 2022) forming three‐dimensional complex habitats on temperate rocky reefs worldwide. These seaweeds form productive and extensive habitats, with their net primary production per unit area approaching values produced by rain forests (Duarte et al., 2022) and contributing ecosystem services to humans (Feehan et al., 2021). Like other ecosystems on land and throughout our oceans, seaweed systems are in rapid transformation globally, with climate‐driven regional or local scale declines playing an increasingly important role in altering seaweed assemblages (de Bettignies et al., 2018; Pecl et al., 2017; Wernberg et al., 2016) and driving corresponding cascading ecosystem‐level effects (Wernberg et al., 2024). Australia is a global seaweed biodiversity hotspot, hosting >350 species of brown seaweeds (class Phaeophyceae; Huisman, 2000). Like many other locations, research into seaweed genetics, ecology, and declines within Australia has focused on the “kelp” species (order Laminariales), which occupy more than one‐third of the world's coastlines (Jayathilake & Costello, 2021). In comparison, relatively few studies have focused on fucoid seaweed taxa (order Fucales; Thomsen et al., 2024), even though their forests are also extensive and serve similar ecological importance and provide food, habitat, and shelter for numerous organisms (Coleman & Wernberg, 2017; Fragkopoulou et al., 2022; Pessarrodona, 2022). Fucoids have high species diversity in Australia (Fragkopoulou et al., 2022), including 63 endemic species, but we know very little about the genetics or ecology of most of these, even though they play a role in supporting biodiversity in temperate regions (Coleman & Wernberg, 2017; Guiry, 2012). Several fucoid species have declined over the past 50 years (Coleman et al., 2008; Coleman & Wernberg, 2017; Smale & Wernberg, 2013), particularly in response to anthropogenic stressors such as ocean warming (Pessarrodona & Grimaldi, 2022), with some species becoming functionally extinct (Pessarrodona, 2022) or experiencing range contractions (Smale & Wernberg, 2013). In this context, understanding the mechanisms of vulnerability and resilience in seaweed populations is of critical importance, but knowledge remains limited.

*Cystophora* (order Fucales) is the second most speciose genus of fucoids globally (Guiry & Guiry, 2025). However, despite their widespread distribution on temperate reefs, there is still minimal understanding of their ecology and genetics (Shepherd & Edgar, 2013). *Cystophora* spp. are endemic to Australia and New Zealand, with some 23 species known from the region (Huisman, 2000), of which 16 occur in Western Australia (Pessarrodona, 2022). *Cystophora* spp. are particularly understudied, with most of the research focusing on the life history and ecology in the central (cooler) portion of the range in South Australia (Collings & Cheshire, 1998; Shepherd & Womersley, 1981), and virtually nothing is known about the genetic diversity within and among species. *Cystophora* spp. are vulnerable to climatic pressures such as increasing ocean temperatures (Pessarrodona, 2022), with documented functional extinctions having recently occurred across portions of the geographic range (Pessarrodona, 2022; Pessarrodona & Grimaldi, 2022; Womersley, 1964). It is important to develop an understanding of how this group of species respond and adapt to warming, as projections indicate a 50%–97% distribution loss under warmer ocean temperatures associated with future climatic scenarios (Martínez et al., 2018; Pessarrodona & Grimaldi, 2022), making species in the *Cystophora* genus ideal candidates for studying climate‐driven genetic shifts and adaptation. To do this, it is particularly important to understand signatures of local adaptation and identify unique genetic variation across species' geographic ranges, with such genomic knowledge potentially helping to inform management practices, such as through the selection of genomic provenances for conservation and restoration (Coleman et al., 2020; Wood et al., 2019, 2021, 2024).

Here, we fill this knowledge gap by characterizing genetic diversity and structure of both neutral and adaptive loci of one of the dominant canopy‐forming fucoid seaweed species in Western Australia, *Cystophora racemosa*. *Cystophora racemosa* was selected for this study due to its ecological importance as a dominant canopy‐forming species in Western Australia (Pessarrodona, 2022; Pessarrodona & Grimaldi, 2022), playing a key ecological role and spanning environmental gradients that allow for the study of patterns of differing genetic diversity and local adaptation. Western Australia provides a particularly unique system for studying these signatures of local adaptation and genetic variation due to its distinct oceanographic features. The Leeuwin Current, a warm, southward‐flowing current, plays a crucial role in shaping the distribution and connectivity of marine populations along the coast (Akhir et al., 2020; Pearce & Pattiaratchi, 1999). In contrast, the Capes Current and other regional hydrodynamic processes create complex environmental gradients that can drive local adaptation (Akhir et al., 2020; Pearce & Pattiaratchi, 1999). These unique oceanographic conditions, combined with the region's high endemicity and historical isolation (Phillips, 2001), make Western Australia an ideal setting for investigating how genetic diversity and adaptation are influenced by environmental variability. Understanding *C*. *racemosa*'s genetic diversity and structure is essential for predicting its potential for resilience and adaptation under climate change. Our study had three aims: (1) to identify any putative signatures of local adaptation and better understand the relative contribution of geographical and environmental drivers on population genetic patterns covering a ~850 km longitudinal portion of the species' range in Western Australia, (2) to assess patterns of neutral and adaptive genetic diversity and genetic structure across populations, and (3) to provide a first step in the assessment of an ecologically important, endemic fucoid and inform future management of *C. racemosa* under a changing climate.

## MATERIALS AND METHODS

### Study species

**FIGURE 1:**
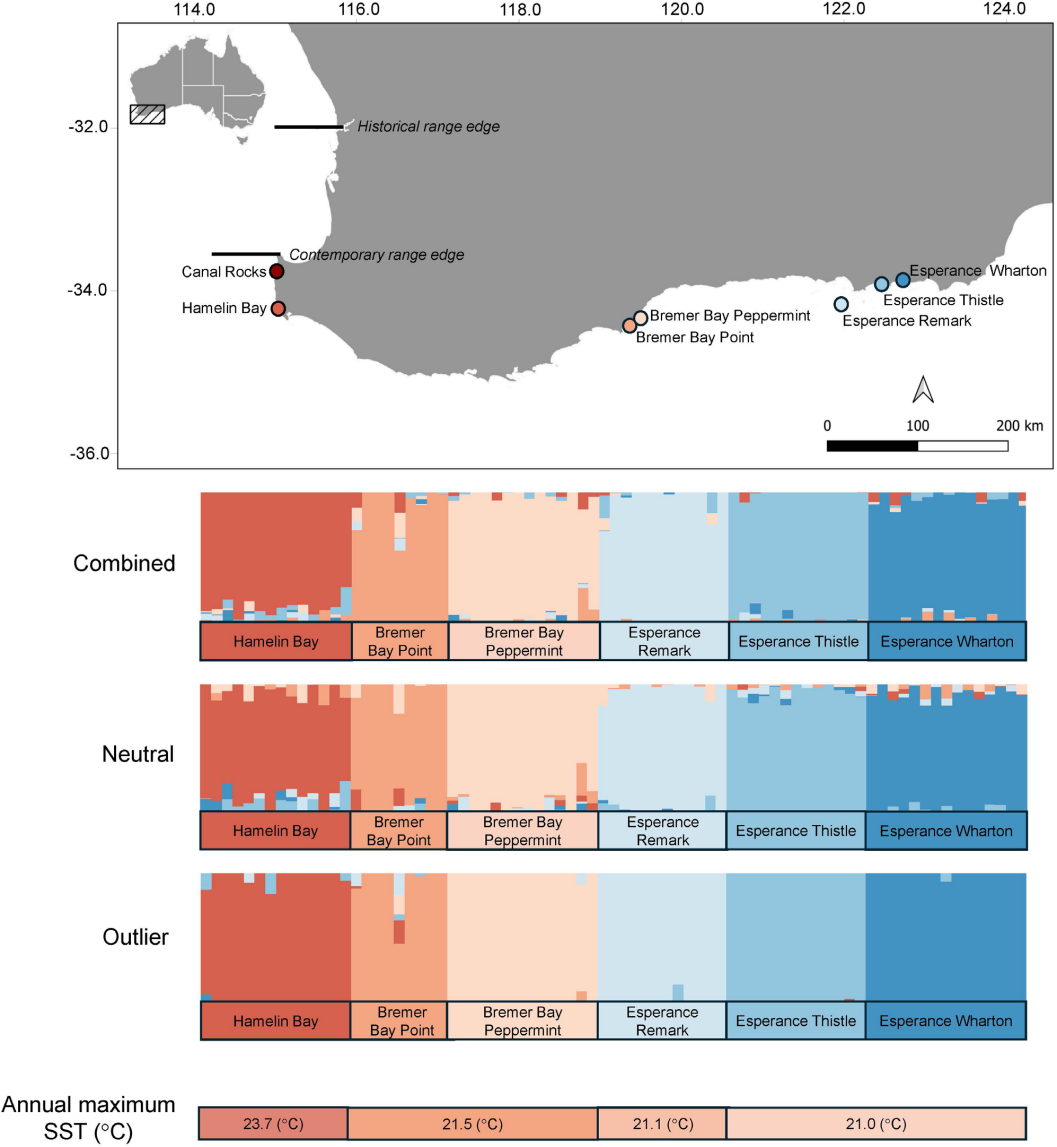
Sampling locations and admixture results of cross‐entropy (CE) clustering inferred with sNMF for *Cystophora racemosa* computed on combined loci dataset (*n* = 76 individuals, 4741 SNPs), neutral loci dataset (*n* = 76 individuals, 4217 SNPs), and outlier loci dataset (*n* = 76 individuals, 70 SNPs). Each bar corresponds to a *C. racemosa* individual, with shared color indicating genetic homogeneity and membership proportion of each cluster, inferring ancestry. Annual maximum SST (°C) for each location presented at the bottom. Historical and contemporary range edges are noted on the map.

*Cystophora racemosa* (order Fucales) is a monoecious seaweed that forms mixed canopies on reefs across southern Australia, from the intertidal zone down to a depth of 14 m (Goldberg & Kendrick, 2004; Pessarrodona & Grimaldi, 2022). Reproductive individuals produce numerous receptacles that contain bisexual or unisexual conceptacles, with antheridia and oogonia being produced in conceptacles, and reproduction occurring via sexual mechanisms (Kabir, 2016; Womersley, 1964). Heightened climatic pressures have restricted the remaining populations to cool, southern waters from Geographe Bay (Western Australia) to Queenscliff (Victoria, Australia) along Australia's Great Southern Reef (GSR; Pessarrodona, 2022; Pessarrodona & Grimaldi, 2022; Figure 1). The western portion of the geographic range of *C. racemosa* in Western Australia encompasses a longitudinal (east to west) temperature gradient. The Leeuwin Current is the dominant boundary current that spans the region; however, the strength of the current is predicted to lessen under future climate scenarios (Akhir et al., 2020; Pearce & Pattiaratchi, 1999). This region is expected to undergo significant warming in the future, with *Cystophora* spp. predicted to lose >70% of their current distribution (Martínez et al., 2018). A combination of surveys and comparisons with historical data has indicated that there has been a significant range contraction of *C. racemosa* in Western Australia, at its contemporary rear edge around the Perth Metropolitan Area (~32° S), with losses at the warm‐range edges occurring in the early 2000s (Pessarrodona, 2022; Pessarrodona & Grimaldi, 2022).

### Field sampling and genetic processing

We sampled individuals at seven sites across ~850 km of coastline in Western Australia (Figure 1). Within each site, *n* = 20 individuals were sampled haphazardly at 6–12 m depths, with individuals collected more than 2 m apart by divers on SCUBA who were familiar with the identification of *Cystophora racemosa*. The sampled sites fell within three broad regions: (1) Capes Region (Canal Rocks and Hamelin Bay), (2) Bremer Bay Region (Bremer Bay Point and Bremer Bay Peppermint), and (3) Esperance Region (Esperance Remark, Esperance Thistle, and Esperance Wharton); however, all sites were treated independently for all analyses. Small parts (ca. 10 separate pieces) of ramuli tissue near the meristem on the primary lateral (ca. 0.5–5 cm long) were selected for genetic processing. The tissue was cleaned of epiphytes by gentle scraping using a sterile scalpel blade, rinsed in fresh water, and blot dried, then snap‐frozen in liquid nitrogen and stored at −80°C until further processing. Individuals were freeze dried for 72 h, and 10–15 mg of tissue per sample was processed for DNA extraction by Diversity Arrays Technology Pty Ltd. (DArT; see details in Kilian et al., 2012) using a modified plant DNA extraction protocol. The individuals were genotyped using DArTseq technology, whereby next generation sequencing techniques are used to identify single nucleotide polymorphisms (SNPs; Pontes et al., 2015). A total of between 10 and 15 individuals per site and one technical replicate (sequenced twice, once in each plate) were sequenced across two sample plates on the Illumina HiSeq 2500 sequencer by DArT in December 2023.

### SNP calling and raw data filtering

Generated SNP variants were processed using proprietary DArT analytical pipelines. For more details, see DArT Pty Ltd.'s website (https://www.diversityarrays.com/technology‐and‐resources/dartseq/), as well as procedures outlined in Sansaloni et al. (2011) and Lind et al. (2017). *Cystophora racemosa* is a non‐model species; therefore, reference alleles and SNP alleles were assigned de novo using a hard‐call approach. Single nucleotide polymorphisms were scored as codominant markers with 0 used when homozygous for the reference allele, 1 when heterozygous, and 2 when homozygous for the alternative allele. The raw data consisted of 98 individuals (17,239 SNPs), 23.8% mean missingness, and a mean read depth of 6.63. To retain only high‐quality SNPs, we filtered the raw data with the dartR package in R version 2.9.7 (Gruber et al., 2018). We initially investigated the effect of different filtering parameters on population genetic diversity metrics (Table S1) to ensure that genetic diversity estimates were accurate and not influenced by excessive missing data among populations (Table S2), read depth, and other filtering parameters. Following the exploration of different filters, we filtered SNPs based on the following criteria to maximize the number of SNPs retained while ensuring that the loci included in the analysis had acceptable levels of reproducibility and low missing data rates: (1) minimum read depth of 2× and maximum read depth of 12×, (2) maximum missingness of 35% among individuals, and (3) maximum missingness of 20% among SNP loci. The reproducibility threshold (the proportion of times the same genotype is called across replicate samples) was set to default value of 0.96 (Gruber et al., 2018), along with a minor allele frequency (MAF) value of >1%. Monomorphs and secondaries (loci that come from the same fragment or sequence as another SNP) were removed to account for linkage disequilibrium, and loci were assessed for significant deviation (*p* < 0.05) from the Hardy–Weinberg Equilibrium (HWE) using Bonferroni's adjustment, with none deviating. The filtered dataset contained 76 individuals and 4741 SNPs, of which mean missingness per individual ranged between 5.22% and 11.04% across populations (Table S2). Only two individuals from the site “Canal Rocks” passed filtering due to high missingness (Table S2), and therefore, we omitted the site from further analyses. Single nucleotide polymorphism error rate between technical replicates was assessed (Mastretta‐Yanes et al., 2015) and determined to be 1.85% in this study.

### Outlier loci detection

#### Environmental data

We were particularly interested in sea surface temperature (SST, °C) as a potential environmental driver of natural selection due to its known association with the distribution of *Cystophora racemosa* and vulnerability to decline (Martínez et al., 2018; Pessarrodona & Grimaldi, 2022). We extracted values for annual maximum SST (°C; Table S3) and other biologically relevant environmental variables (current velocity, nitrate, salinity, and light) for the sampled sites using Bio‐ORACLE version 2.0 (Assis et al., 2018). Current velocity influences propagule dispersal, nutrient transport, and larval connectivity (Gaylord et al., 2012), with dispersal potential dictating gene flow and colonization success (Coleman et al., 2011). Nitrate, as a key macronutrient, influences algal growth and physiological responses, potentially driving differences in fitness in nutrient‐limited environments (Rugiu et al., 2020). Salinity directly affects cellular osmoregulation and metabolic function, and variations in salinity across sites may exert selective pressures that shape genetic adaptations enabling populations to persist under specific salinity regimes (Roleda & Hurd, 2019). Light availability affects photosynthetic efficiency, which in turn impacts productivity and energy allocation (Cronin & Hay, 1996). Environmental variables were assessed for (i) variation across sites and (ii) redundancy using Pearson's correlation coefficient in R (Table S4). Following screening, only the annual maximum SST (°C) variable was selected for analysis, as other variables did not vary at the relevant spatial scales and/or were highly correlated (Pearsons's correlation coefficient > 0.7 or <−0.7; Table S4).

#### Outlier detection methods

Due to high false positive rates surrounding detecting outlier SNPs (putative loci under selection; O'Leary et al., 2018; Phair et al., 2019), four methods were used to identify outlier loci on the combined loci dataset (35% maximum missingness allowed; 4741 SNPs) and were subsequently compared: BayeSCAN v.2.1 (Foll & Gaggiotti, 2008), Pcadapt (Luu et al., 2017), latent factor mixed models (LFMM; Frichot et al., 2015) and a redundancy analysis (RDA; Oksanen et al., 2019). Two of the methods (LFMM and RDA) are Genotype‐Environment Association (GEA) methods and use allele frequency correlations with environmental variables (annual maximum SST) to identify candidate outlier loci. BayeSCAN uses a posterior probability value for each SNP locus based on the observed *F*_ST_ distribution, with outlier loci identified as those that deviate from neutral expectations (Foll & Gaggiotti, 2008), and PCadapt identifies outliers with respect to how they are related to population structure as ascertained by principal component analyses (Luu et al., 2017).

BayeScan was run for 10 runs on default parameters with 20 pilot runs of 50,000 iterations, with a false discovery rate (FDR) threshold of 0.05, prior odds of 10, and a 10% burn‐in period. Using BayeScan, SNPs were categorized as balancing, diversifying, and neutral (Foll & Gaggiotti, 2008). If ALPHA was ≥0 and *q*‐value was ≤0.05, then the loci were defined as being under potential “diversifying selection,” whereby the *q*‐value is the minimum FDR at which the test result can be called significant (Lai, 2017). If ALPHA was ≥0 and *q*‐value was ≥0.05, then the loci were defined as being under potential “neutral selection,” and if neither of the above conditions were met, then the loci were defined as being under potential “balancing selection.” PCadapt was implemented using the R package pcadapt (Luu et al., 2017), and candidate outlier SNPs were defined as having a *q*‐value of less than 0.01, bounded by an FDR of 0.01 on the first two principal components. Latent factor mixed models were implemented using the R package LEA (Frichot et al., 2015). Missing allele frequencies were imputed using the number of ancestry coefficients estimated by sNMF (distinct genetic clusters, *K* = 6), identified by 10 runs at an alpha value of 100 based on cross‐entropy criterion. The sNMF function assumes that genetic data originates from the admixture of *K* parental populations, where *K* is unknown (Frichot et al., 2014). An estimate of ancestry proportions for each multilocus genotype was computed, and a cross‐entropy plot was visualized to inform the best choice for the number of *K* parental populations. A Markov chain Monte Carlo algorithm was used to estimate latent factors and effect sizes for the environmental variable annual maximum SST (°C), which was used to compute *z*‐scores using 50,000 steps for burn‐in and an additional 100,000 steps. All *p*‐values were adjusted to <0.01 using the Benjamini–Hochberg procedure, and the genomic inflation factor (GIF) value was calculated and manually adjusted (0.9) to modify the *z*‐scores to control for FDR (Frichot et al., 2015; Figure S1). An FDR of 0.01 was then applied to identify candidate outlier SNPs. Lastly, an RDA was implemented using the R package vegan (Oksanen et al., 2019). The RDA is also a GEA method; however, it is a multivariate method that simultaneously analyzes multiple loci to detect signatures of multilocus selection pressures (Forester et al., 2018; Waters et al., 2013). Annual maximum SST (°C) was included as the explanatory variable in the analysis, and allelic frequencies generated from sNMF were imputed. Finally, the significance of the RDA axis was assessed using an ANOVA with 9999 permutations, and loci with a loading greater than ±3.0 *SD* (two‐tailed *p*‐value = 0.0027) were considered as outliers (Figure S2).

Outlier loci identified by at least two of the methods (Figure S3) were considered to be under putative selection and isolated from the combined SNP dataset to create an “outlier loci dataset.” All outlier loci identified across the four methods were also removed from the combined dataset to create a “neutral loci dataset.” All subsequent analyses were run on two datasets—neutral SNPs and outlier SNPs; however, additional structure analyses were performed on the combined loci dataset to investigate overall population genetic structure. Missingness of each outlier loci was calculated as a mean value across all individuals (mean = 6.17%) and ranged from 0.00% to 19.80% missingness across sites (Figure S4). Missingness was also calculated for outlier and neutral loci within sites (Table S5); however, there were no differences between sites. To further assess whether imputation influenced our results, we calculated allele frequencies for loci identified as outliers in RDA and LFMM (*n* = 14 SNPs) and examined their distributions across populations (Figures S5 and S6). This analysis confirmed that imputation was not a driving factor in detecting loci under selection, as allele frequency patterns were consistent with population structure rather than being artifacts of missing data.

### Population genomic structure

A principal coordinates analysis (PCoA) was used to visualize the spatial relationships among all sampled individuals in the (1) combined loci, (2) neutral loci, and (3) outlier loci datasets and using the gl.pcoa function (Gruber et al., 2018). Identification of the number of ancestral populations and degree of admixture was investigated for all three datasets using non‐negative matrix factorization algorithms, computed on least‐squares estimates of ancestry coefficients of populations using the sNMF function (Frichot et al., 2014).

### Isolation by distance

Pairwise oceanographic distance between sampling sites was computed in the R package melfuR (Brauer, 2019; Table S6). For both the neutral loci and outlier loci, a Mantel test was run with 9999 permutations to investigate the effects of oceanographic distance on Euclidean genetic distance between sites (i.e., isolation by distance and IBD) in the adegenet R package (Jombart, 2008). A Pearson's correlation coefficient (*r* ^2^) was used to assess the strength of the correlation between distance matrices.

### Population genomic diversity

Population diversity statistics were calculated on the neutral and outlier loci separately. Diversity statistics included the number of private alleles in the population (*N*_P_), allelic richness (*A*_R_), observed (*H*_O_) and expected heterozygosity (*H*_E_), and inbreeding coefficient (*F*_IS_). The number of private alleles (private) was computed using the private_alleles function (Kamvar et al., 2014), and rarefied allelic richness (AR) was calculated using the allelic.richness function (Goudet, 2005). Observed (*H*_O_), expected heterozygosity (*H*_e_), and the inbreeding coefficient (*F*_IS_) were calculated using the gl.report.heterozygosity function (Gruber et al., 2018), where negative *F*_IS_ values indicate outbreeding and positive *F*_IS_ values indicate inbreeding. Pairwise *F*_ST_ values were calculated between populations and tested for Bonferroni‐corrected significance (*p <* 0.05) using 999,999 permutations in the STAMPP package (Pembleton et al., 2013), with larger values indicating high genetic differentiation. A hierarchical analysis of molecular variance (AMOVA) was computed using the poppr function (Kamvar et al., 2014), partitioning the percentage of genetic variation attributed within and between populations.

## RESULTS

### Identification of outlier and neutral loci

The “combined loci” dataset composed of a total of 4741 SNPs (*n* = 76 individuals) was used for outlier detection. A total of 524 “outlier” SNP candidates (11% of the total combined loci dataset) were identified across all four statistical methods (Figure S3). The RDA identified the most outlier SNPs (*n* = 320, 6.8%), followed by PCadapt (*n* = 191; 4.1%), LFMM (*n* = 72, 1.5%), and BayeScan (*n* = 32, >1%; Figure S3). A handful of SNPs (*n* = 8) were shared between all methods, while 70 (1.5%) were identified by at least two methods. These 70 SNPs were subsequently used to form the “outlier loci” dataset. All outlier SNPs identified across the four methods were removed from the combined loci dataset to form the “neutral loci” dataset (*n* = 4217 SNPs). Outlier SNPs detected by only one method were therefore removed from both neutral and adaptive datasets. Out of the 32 SNPs identified in BayeScan, 18 SNPs were under potential balancing selection, and 14 SNPs were under potential diversifying selection.

### Population structure and isolation by distance

Population structure was clearly defined for the combined loci dataset (both neutral and outlier loci; *n* = 76 individuals 4741 SNPs; Figure 1). The cross‐entropy (CE) plot revealed that *K* = 6 had the lowest CE value. Thus, we inferred a total of six ancestral populations (i.e., one for each sampled site). The PCoA supported the structure analysis, revealing a clear separation between all sampled sites (Figure S7).

**FIGURE 2:**
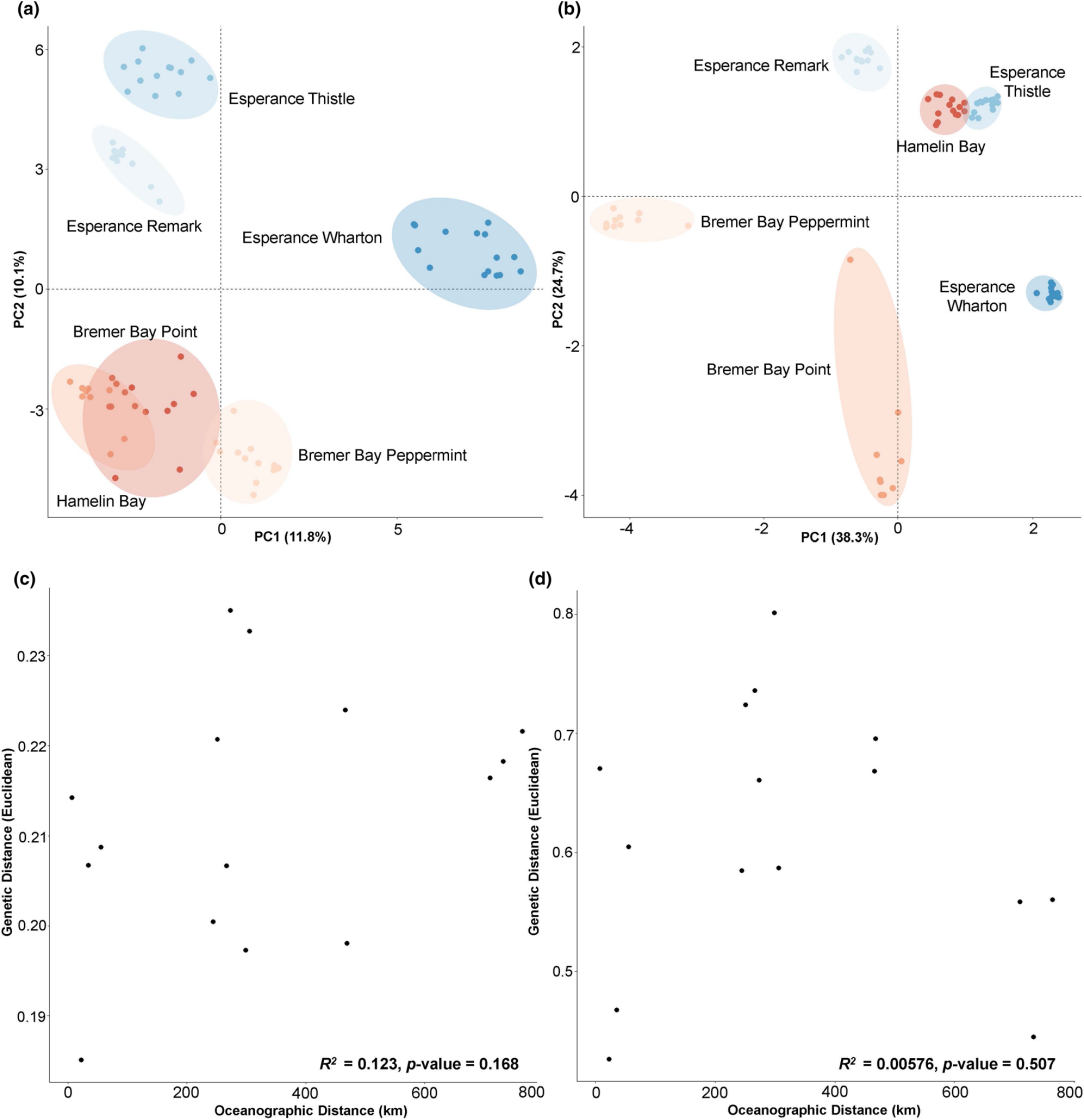
Principal Coordinates Analysis (PCoA) of the (a) neutral dataset (*n* = 76 individuals, 4217 SNPs) and (b) outlier dataset (*n* = 76 individuals, 70 SNPs). Different colors represent different sites, with points corresponding to individuals. Isolation by distance using Oceanographic Distance (km) and Genetic Distance (Euclidean) for the (c) neutral dataset (*R* ^2^ = 0.123, *p*‐value = 0.168) and (d) outlier dataset (*R* ^2^ = 0.00576, *p*‐value = 0.507).

**FIGURE 3:**
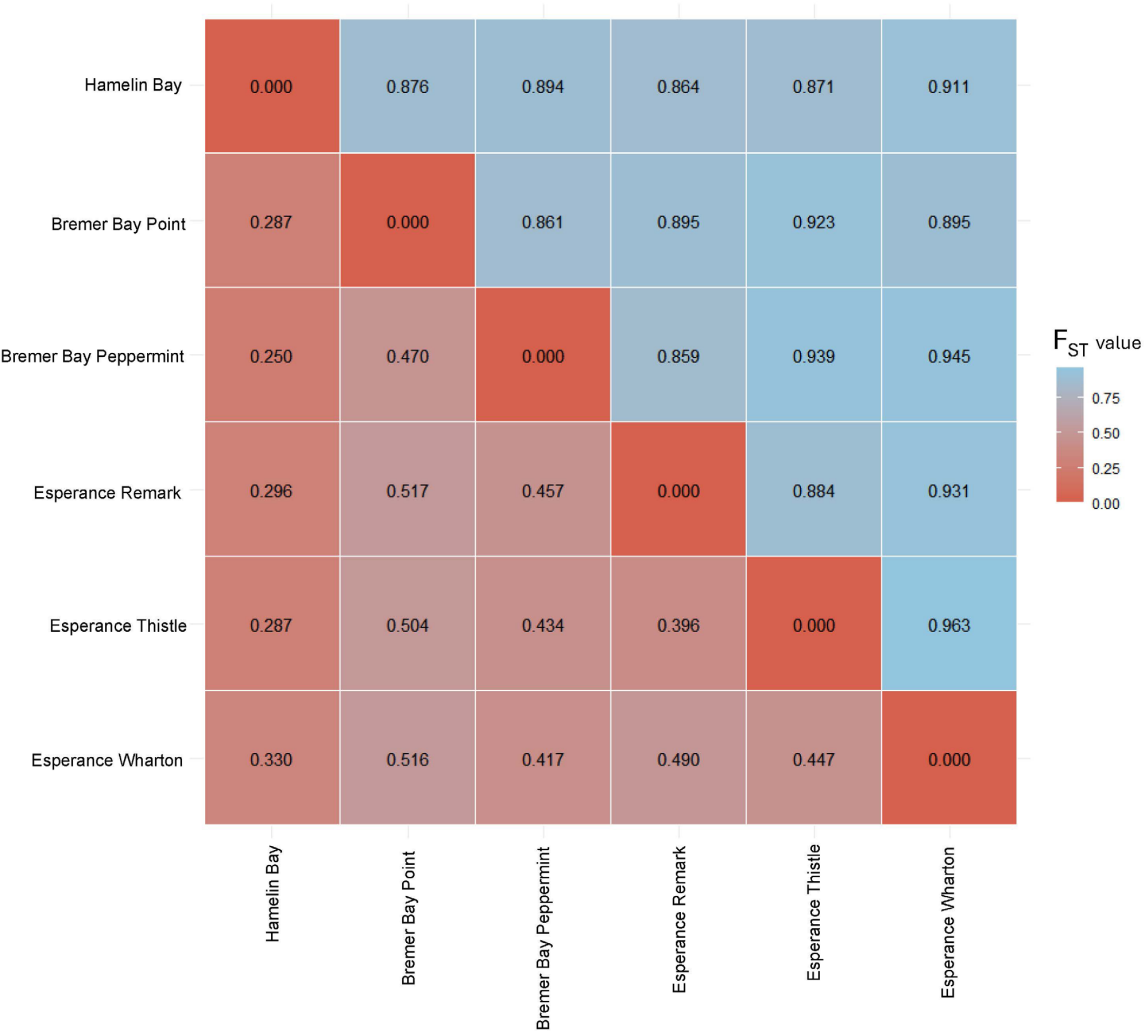
Genetic differentiation indicated by pairwise *F*_ST_ between sites corrected with Bonferroni adjustment, based on neutral loci dataset (*n* = 76 individuals, 4217 SNPs; red lower half) and outlier loci dataset (*n* = 76 individuals, 70 SNPs; blue upper half). All values are significant (*p*‐value < 0.05).

In both the neutral and outlier loci datasets, the PCoA and sNMF structure analyses demonstrated clear separation of all sampled sites (*K* = 6 across 6 sites, Figures 1 and 2). Genetic differentiation across sites was high for neutral (mean *F*_ST_ = 0.404) and outlier loci (mean *F*_ST_ = 0.901), with all sites significantly different from one another for both neutral and outlier loci (*p*‐value < 0.05 in both datasets, Figure 3). The hierarchical AMOVA revealed the most genetic variation to be explained by site for neutral loci (38% variation explained between sites), with a significant result based on the *F*‐statistic (*F* = 131.83; *df* = 5 between sites, *df* = 70 between individuals within sites; *p*‐value = 0.001; Table 1). Similarly, the majority of adaptive genetic variation was explained by site and was particularly high (87% variation explained between sites, *F* = 11.18; *df* = 5 between sites, *df* = 70 between individuals within sites; *p*‐value = 0.001; Table 1). Surprisingly, there was no evidence of isolation by distance in either the neutral or outlier datasets, based on Mantel tests (neutral: *R* ^2^ = 0.123, *p*‐value = 0.168; outlier: *R* ^2^ = 0.00576, *p*‐value = 0.507; Figure 2). However, in the neutral dataset, some nearby sites did cluster near each other (e.g., Bremer Bay sites; Figure 2).

**TABLE 1:** Hierarchical analysis of molecular variance (AMOVA) of the neutral dataset (*n* = 76 individuals, 4217 SNPs) and outlier dataset (*n* = 76 individuals, 70 SNPs).

	*df*	Mean Sq	σ	%	*p*
Neutral					
Between sites	5	1515.699	54.832	38.444	0.001
Between individuals within sites	70	132.788	443,991	31.544	0.001
Within individuals	76	42.806	42.806	30.012	0.001
Total	151	133.291	142.629	100.000	
Outlier					
Between sites	5	313.612	12.342	87.396	0.001
Between individuals within sites	70	2.332	0.552	3.913	0.001
Within individuals	76	1.227	1.227	8.691	0.001
Total	151	12.084	14.122	100.000	

*Note*: Percent of variance of the total (%) for each hierarchical level and *p*‐value (*p*).

Abbreviations: σ, variance; *df*, degrees of freedom; Mean Sq, mean of squares.

Interestingly, the PCoA analysis revealed that patterns of genetic similarity across sites differed between outlier and neutral loci datasets (Figure 2). In the neutral dataset, Hamelin Bay clustered with the Bremer Bay sites, but in the outlier dataset, Hamelin Bay clustered with Esperance Thistle. Although the clustering of Hamelin Bay with the Bremer sites may reflect a weak effect of oceanographic distance, it is less clear why Esperance Thistle and Hamelin Bay would share similar allele frequencies at putatively adaptive loci. Given that these sites differ in sea surface temperature, we hypothesize that these sites may be experiencing parallel selection for annual maximum SST (°C) and non‐temperature correlated variables such as light, pH, or salinity that we were unable to capture in this study.

### Population genetic diversity

For the neutral dataset, heterozygosity estimates were relatively low across all sites (mean ± *SD*, *H*_E_ = 0.046 ± 0.021, range 0.028–0.091), with the most western population, Hamelin Bay, showing the highest heterozygosity estimates out of all sites (*H*_E_ = 0.091; Table 2). Private neutral alleles were overall high and located in all sites, ranging from 304 (7.2% of total SNPs) at Bremer Bay Point to 1088 at Hamelin Bay (25.8% of total SNPs; Table 2). High levels of inbreeding were observed across all sites (*F*_IS_ = 0.531 ± 0.113, 0.336–0.688) and allelic richness showed similar trends to expected heterozygosity across all sites (A_R_ = 1.046 ± 0.022, 1.029–1.095).

**TABLE 2:** Genetic diversity metrics for *Cystophora racemosa* at six sites in Western Australia for the neutral (*n* = 76 individuals, 4217 SNPs) and outlier dataset (*n* = 76 individuals, 70 SNPs).

Site	Latitude	Longitude	*n*	Neutral					Outlier			
				*N*_P_	*A*_R_	*H*_O_	*H*_E_	*F*_IS_	*N*_P_	*A*_R_	*H*_O_	*H*_E_
Hamelin Bay	−34.263	115.024	14	1088	1.095	0.042	0.091	0.562	5	1.052	0.021	0.050
Bremer Bay Point	−34.400	119.410	10	304	1.038	0.025	0.036	0.336	6	1.087	0.032	0.082
Bremer Bay Peppermint	−34.400	119.480	12	344	1.043	0.022	0.041	0.494	9	1.067	0.053	0.065
Esperance Remark	−34.045	121.991	12	308	1.029	0.015	0.028	0.479	0	1.045	0.024	0.043
Esperance Thistle	−34.000	122.204	13	557	1.042	0.013	0.040	0.688	0	1.003	0.003	0.003
Esperance Wharton	−33.944	122.559	15	417	1.041	0.015	0.039	0.625	0	1.012	0.006	0.011

Abbreviations: *A*_R_, rarefied allelic richness; *F*_IS_, inbreeding coefficient; *H*_E_, expected heterozygosity; *H*_O_, observed heterozygosity; *n*, number of individuals genotyped; *N*_P_, number of private alleles.

Genetic diversity parameters for the outlier dataset showed distinct patterns from the neutral dataset (Table 2). A similar range in heterozygosity estimates was observed across all sites (*H*_E_ = 0.042 ± 0.028, 0.003–0.082), with the peak adaptive diversity present in Bremer Bay Point (*H*_E_ = 0.171; Table 2). Not surprisingly, fewer private alleles were observed across the sites, with Bremer Bay Peppermint having the highest number of private alleles (*n* = 9) and the Esperance sites having no private alleles (Table 2). Allelic richness showed similar trends to expected heterozygosity across all sites (A_R_ = 1.044 ± 0.029, 1.003–1.087).

## DISCUSSION

Here, we have revealed potential signatures of local adaptation in the ecologically important, climate‐threatened fucoid, *Cystophora racemosa*, with several genetic outlier loci significantly associated with annual maximum SST (°C). Relatively high genetic differentiation between populations and low heterozygosity estimates across sites were observed for both neutral and adaptive loci, and no significant patterns of isolation by distance were observed for either dataset. Critically, our results suggest that there is unique genetic diversity present at fine scales along the south coast of Western Australia. This has important implications for developing conservation and management practices to ensure that these critical *Cystophora* forests are resilient in the face of future environmental change.

### Low levels of within‐population genetic diversity and high levels of inbreeding

Regardless of whether analyzing neutral or adaptive loci, our results consistently revealed low levels of within‐population genetic diversity, as indicated by low heterozygosity for both the neutral (mean ± *SD*, *H*_E_ = 0.046 ± 0.021, range 0.028–0.091) and outlier loci (*H*_E_ = 0.042 ± 0.028, 0.003–0.082). These estimates were lower than those from other SNP‐based studies on fucoids, including for *Ericaria zosteroids (H*_E_ = 0.105 ± 0014; Reynes et al., 2021) and *Phyllospora comosa* (*H*_E_ = 0.278 ± 0.058; Wood et al., 2021), with comparable results to other fucoid species using different molecular markers, such as allozymes for *Pelvetia fastigiata* (*H*_E_ = 0.042 ± 0.032; Williams & Di Fiori, 1996) and microsatellites for *Fucus guiryi* (*H*_E_ = 0.018 ± 0.025; Almeida et al., 2017), as well as some other brown algal groups surveyed using DArTseq methods such as *Nereia lophocladia* (*H*_E_ = 0.055 ± 0.014; Mamo et al., 2021). The observed diversity values were also lower than those for the co‐existing Laminarian species *Ecklonia radiata* across the same sampled range using similar genomic SNP data (*H*_E_ = 0.138; Vranken et al., 2021). These lower observed heterozygosity estimates for both neutral and outlier loci could possibly be attributable to technical differences in the generation of genomic data or to biological processes such as inbreeding (Bein et al., 2023; Olsen et al., 2020). Indeed, our results provided strong evidence for inbreeding (mean ± *SD F*_IS_ = 0.531 ± 0.113, range 0.336–0.688), comparable to other fucoids, for example, *Fucus spiralis* (*F*_IS_ = 0.557 ± 0.113; Perrin et al., 2007) and *Fucus guiryi* (*F*_IS_ = 0.979 ± 0.037; Lourenço et al., 2016). High inbreeding rates are commonly observed in sessile and sedentary species, due to reduced dispersal capacity of gametes and spores, as settled individuals have a reduced opportunity for mating with distant counterparts (Kinlan & Gaines, 2003; Santelices, 1990). Moreover, the life histories of many fucoids may promote inbreeding, including selfing (reviewed in Hatchett et al., 2022). Given that many fucoid species are monoecious (Coleman et al., 2019; Coleman & Wernberg, 2017; Hatchett et al., 2022), the higher potential for self‐fertilization may further contribute to the observed levels of inbreeding and low genetic diversity (Coleman & Brawley, 2005). The increased proximity of related individuals can exacerbate inbreeding effects over time (Krueger‐Hadfield et al., 2015; Riquet et al., 2021). Monoecy thus likely plays a role in shaping the observed genetic patterns in *Cystophora*, contributing to its low heterozygosity and high inbreeding coefficients.

In many fucoid species, over 90% of the propagules settle within meters of the parental thalli (Kendrick & Walker, 1995), with long‐distance dispersal possible, mainly by broken‐off and drifting reproductive tissue. Thus, inbreeding may be common across much of the genus. Our study species, *Cystophora racemosa*, has bisexual conceptacles (Womersley, 1964), which can favor self‐fertilization and can lead to higher levels of observed inbreeding than in species with pure unisexual conceptacles. *Cystophora racemosa* has small gas‐filled vesicles on the thallus (Womersley, 1964); therefore, long‐distance dispersal of fragmented thali is likely possible and more common than in other species across the genus that lack vesicles. Our results further demonstrated that despite the presence of inbreeding and low diversity estimates, there was still a high proportion of private alleles present across all sites for the neutral dataset, specifically in Hamelin Bay (25.8% of total SNPs). Private alleles are a measure of genetic distinctiveness (Antonovic, 1990) and potentially contribute to the strong observed genetic differentiation across sites. Hamelin Bay, located near the species' range edge, has been experiencing its highest temperatures, yet it has retained a high number of private alleles. This suggests that although environmental stressors can reduce genetic diversity, Hamelin Bay may not yet have undergone a strong selective bottleneck or passed a critical threshold leading to diversity loss. Instead, it is possible that historical connectivity, genetic drift, or localized adaptation have maintained or even promoted genetic distinctiveness in this population.

The observed genetic diversity and inbreeding estimates were also influenced by the type of markers used in our study. We employed SNP markers generated through DArTseq, a reduced‐representation sequencing approach that samples a subset of the genome (Kilian et al., 2012). Single nucleotide polymorphisms are generally biallelic, which can limit their ability to detect high heterozygosity levels compared to microsatellite markers, which are multiallelic and generally have higher allelic diversity per locus (Selkoe & Toonen, 2006). Although SNPs provide high‐resolution population structure and genome‐wide genetic differentiation estimates, their ability to detect fine‐scale inbreeding levels may be lower than that of microsatellites (Coates et al., 2009; Zimmerman et al., 2020). For comparison, previous studies on fucoid species using microsatellites have reported similar trends of high inbreeding (e.g., *Sargassum horneri* in Wang et al., 2019; *Fucus* spp. in Almeida et al., 2017; and *Phyllospora comosa* in Wood et al., 2021) but often with higher absolute heterozygosity estimates due to the greater variability of these markers. However, SNPs offer a more unbiased assessment of genome‐wide variation and allow for the detection of potential selection signals, making them particularly valuable for studying adaptation in addition to neutral processes.

### Drivers of high differentiation between populations

Genetic differentiation among sites was very high for both the neutral (mean *F*_ST_ = 0.404) and outlier datasets (*F*_ST_ = 0.901). These levels of genetic differentiation suggest that gene flow among populations has been restricted over both contemporary and evolutionary timescales, even across short distances. Indeed, we identified strong differentiation and unique genetic diversity between the Bremer Bay sites, which were only 6.7 km apart (neutral pairwise *F*_ST_ = 0.470). The strong genetic structure observed at neutral loci, which are shaped primarily by historical demographic processes, suggests that restricted gene flow has persisted over extended evolutionary timescales. It is probable that limited gene flow across populations has resulted from a combination of life‐cycle traits that favor limited dispersal (i.e., bisexual conceptacles and low settlement efficiency; Pearson & Serrão, 2006) and changes in environmental conditions due to long‐term historical processes such as changes to climate. Local dispersal can result in fine‐scale population structure (Neiva et al., 2012; Riquet et al., 2021; Tatarenkov et al., 2007), with occasional long‐distance dispersal via vesicle‐mediated rafting enabling rare genetic exchange between distant populations (Coleman et al., 2019; Fraser et al., 2022; Nikula et al., 2013). These dispersal strategies operate at different scales, with limited local dispersal reinforcing genetic structure and promoting inbreeding whereas episodic long‐distance dispersal maintains regional connectivity and introduces genetic variation. The physical configuration of the shoreline itself may also play a role in reinforcing these patterns. Dispersal and gene flow can be limited by coastal geomorphology, habitat discontinuities, and wave exposure (Binks et al., 2019; Hernawan et al., 2023; Johansson et al., 2015). These mechanisms may reduce effective gene flow, even among geographically proximate populations, allowing locally adapted lineages to persist and diverge over time. These patterns also raise the possibility of cryptic speciation, a phenomenon increasingly recognized in fucoid algae (see Almeida et al., 2022; Billard et al., 2010; Zardi et al., 2011). A similar process may be operating within *Cystophora racemosa*, for which life‐cycle traits of limited dispersal and potential self‐fertilization could reinforce reproductive isolation and facilitate the emergence of cryptic lineages. Further genetic and reproductive studies are needed to test this hypothesis and to explore the hidden diversity within the species, particularly for adaptive loci.

One possible hypothesis for the observed high genetic differentiation is that populations were once more connected than they are today, possibly via a rapid colonization event following a warm period that made the region uninhabitable for *Cystophora*. Over the past few hundred thousand years, Australia has been affected by multiple glacial (cold) and interglacial (warm) periods that are linked to global Milankovitch cycles (Barrows et al., 2002; Cadd et al., 2021; Chang et al., 2015; Lewis et al., 2013). During this period, surface and bottom water temperatures fluctuated, which could have impacted the geographical distributions of species. Cooler than present temperatures may have assisted in sustaining the rapid expansion of *Cystophora* across the southern coastline, as cooler temperatures allowed for faster growth and reproduction that facilitated population expansion (Brookes et al., 1992; Myers & Giller, 1988). However, periods of warmer ocean temperatures could have led to the mortality of the species, as their thermal tolerance was exceeded, resulting in the isolation of gene pools, as portions of species ranges were fragmented, causing patchy habitats that led to high genetic differentiation over time. These temperature fluctuations likely also affected the strength of predominant currents (Akhir et al., 2020; Spooner et al., 2011), which may have influenced rates of dispersal via rafting. Assuming a rapid colonization event occurred following climatic fluctuations, it is difficult to disentangle from where the source population of *Cystophora* would have originated, as we do not have data on divergence times between populations. However, it is possible that the source population originated from the southwest coast around the Cape Naturaliste and Cape Leeuwin region. This hypothesis is supported by the observation of the highest genetic diversity at Hamelin Bay, which may have acted as (or been near) a refugial population that survived the most recent of these climate cycles. The Capes region has high genus and species endemism and is thought to have experienced long‐term geological and climate stability (Phillips, 2001). It is possible that the stability in climate has allowed the Hamelin Bay population to evolve in isolation over time and expand during periods of more favorable climatic conditions further east. Interestingly, unique genetic diversity (Sinclair et al., 2023; Vranken et al., 2021) and endemic species (Coleman & Wernberg, 2018; Kuo & Cambridge, 1984) have been observed in the Capes region around Hamelin Bay for other marine taxa, possibly attributable to the historical processes that have created geographic and genetic isolation of the region over time.

### Signatures of selection and adaptive genetic structure

We observed different patterns of clustering between the neutral and outlier datasets, possibly hinting at signs of parallel adaptation across different sites (Ralph & Coop, 2010; Wood et al., 2005). Although differences in clustering patterns between neutral and adaptive loci cannot be explained by annual maximum SST (°C), we hypothesize that Esperance Thistle and Hamelin Bay may experience similarities in biologically relevant environmental variables that we were unable to measure. If this is the case, then parallel selection to this latent variable could explain this discrepancy in clustering patterns across neutral and adaptive loci. We recommend experimental manipulation studies be performed on *Cystophora racemosa* to assist in untangling the driving factors behind the observed patterns of putative selection, including the other environmental variables that are ecologically relevant (e.g., nutrients, light, pH, and marine heatwave events). Furthermore, additional genetic sampling is recommended on smaller scales (i.e., meters) to investigate to what degree genetic structure and differentiation become apparent between sites, which will inherently inform management practices.

### Implications for management of *Cystophora racemosa*

High differentiation across populations of *Cystophora racemosa* suggests that there is unique genetic diversity across short distances in southwest Australia. Our study can inform management practices, with an overall aim of ensuring sufficient genetic diversity is present for future adaptation to changing environmental conditions, particularly the projected increase in SST (°C; Coleman et al., 2020; Wood et al., 2019). The results from this study suggest that unique genetic diversity along the southern coastline means conservation and management should encompass populations across the range of the species, not just one area. Given the high genetic differentiation and probable local adaptation identified here, potential genetic‐rescue or assisted‐gene‐flow strategies may be useful methods to enhance the resilience of vulnerable populations facing anthropogenic stressors and continued warming. In particular, the near warm range‐edge site Hamelin Bay has noteworthy conservation value due to the high number of private alleles revealed for the neutral dataset and overall highest genetic diversity for the neutral loci, suggesting it could reflect a historical refugia population that is the original source of recolonizing populations, and may be a useful source for genetic rescue (Whiteley et al., 2015) or targeted assisted gene flow (Aitken & Whitlock, 2013). Such an approach, however, would require additional testing and ground truthing through manipulative experiments that test causational responses to possible selective pressures (Kettenring et al., 2014; Reynolds et al., 2016) and identify potential maladaptation or outbreeding depression effects that may occur when crossing distinct populations (Frankham et al., 2011; Templeton, 1986; Thornhill, 1993). Regardless, Hamelin Bay experiences the warmest annual maximum average SST (°C) out of all sites we examined here and, thus, may be the first where *C*. *racemosa* disappears under warming climatic conditions. Therefore, it is paramount to develop strategies to conserve the unique genetic diversity at this site and elsewhere, for example via acrogenic conservation of germplasm (Daly et al., 2022).

## CONCLUSIONS

We have characterized neutral and adaptive population genomic diversity and structure of the Australian endemic fucoid, *Cystophora racemosa*, across an ~850 km longitudinal range in Western Australia. We identified high genetic differentiation but no evidence of isolation by distance across all sampled populations, including those only a few kilometers apart. We also observed high levels of inbreeding and low population genetic diversity estimates, which suggest that populations may be particularly vulnerable to future climate change unless rapid, widespread gene flow occurs. As environmental degradation and climate change impacts are set to increase, it is paramount to apply knowledge of population genomics, the foundation of evolution and adaptation, to restoration and management practices (Breed et al., 2019; Coleman et al., 2020; Wood et al., 2019). Although our sub‐setting approach helped improve data robustness, it did not fully eliminate the risk of genotyping errors associated with low‐coverage loci. It is important to acknowledge that low‐coverage loci, even after filtering, may still be prone to genotyping errors. Sufficient replication, particularly in studies with limited sequencing depth, is therefore essential for distinguishing true biological patterns from technical noise. Our study provides an important step in this regard, which we hope sets the stage for further efforts to use genomic tools to safeguard these unique marine forests.

## AUTHOR CONTRIBUTIONS

**Jane M. Edgeloe:** Conceptualization (equal); data curation (equal); formal analysis (lead); investigation (lead); methodology (lead); resources (equal); software (equal); validation (lead); visualization (lead); writing – original draft (lead); writing – review and editing (lead). **Samuel Starko:** Formal analysis (supporting); investigation (supporting); methodology (supporting); supervision (supporting); visualization (supporting); writing – original draft (supporting); writing – review and editing (supporting). **Albert Pessarrodona:** Data curation (supporting); funding acquisition (equal); methodology (supporting); writing – review and editing (supporting). **Melinda A. Coleman:** Methodology (supporting); resources (supporting); writing – review and editing (supporting). **Jacqueline Batley:** Writing – review and editing (supporting). **Thomas Wernberg:** Funding acquisition (supporting); resources (supporting); writing – review and editing (supporting). **Georgina V. Wood:** Conceptualization (supporting); data curation (equal); formal analysis (supporting); funding acquisition (lead); investigation (supporting); methodology (supporting); project administration (lead); resources (supporting); software (supporting); supervision (lead); validation (supporting); visualization (supporting); writing – original draft (supporting); writing – review and editing (supporting).

## Supporting information


**Figure S1.** Histograms of test significance values (*p*‐values) for LFMM, using the adjusted genomic inflation factor of 0.9 (GIF) for SST max (°C).
**Figure S2.** Redundancy analysis (RDA) for the combined dataset (4741 SNPs).
**Figure S3.** Putative adaptive loci identified by four methods: BayeSCAN, PCadapt, LFMM and RDA.
**Figure S4.** Missingness per SNP (%) for the neutral (*n* = 4217 SNPs) and adaptive (*n* = 70 SNPs) datasets for all six sites combined.
**Figure S5.** Allele frequencies of adaptive loci identified by RDA and LFMM (*n* = 14 SNPs) for six populations, whereby data is not imputed.
**Figure S6.** Allele frequencies of adaptive loci identified by RDA and LFMM (*n* = 14 SNPs) for six populations, whereby data is imputed by sNMF.
**Figure S7.** Principal Coordinates Analysis (PCoA) of the combined dataset (*n* = 76 individuals, *n* = 6 sites, 4741 SNPs).
**Table S1.** The effect of SNP filtering cut‐offs on the number of individuals (Ind. Count), number of SNP loci (SNP count), missingness (%) per SNP, mean read depth, mean (± *SD*) H_E,_ mean (±*SD*) *F*_IS_, overall (± *SD*) *F*_ST_.
**Table S2.** Number of individuals per site (Ind. Count) before (*n* = 17, 239 SNPs) and after (4741 SNPs) filtering, respective site level mean missing rate on individuals (%) calculated before and after filtering.
**Table S3.** Environmental variables values for six sites, extracted from Bio‐ORACLE version 2.0 (Assis et al., 2018).
**Table S4.** Pearson's correlation coefficient values for pairs of environmental variables extracted from Bio‐ORACLE version 2.0 (Assis et al., 2018).
**Table S5.** Mean missingness of adaptive loci (*n* = 70 SNPs) and neutral loci (*n* = 4421 SNPs) for six sites.
**Table S6.** Pairwise oceanographic distance (km) between sites, calculated in melfuR.

## Data Availability

Genetic data will be deposited on DRYAD after acceptance. https://doi.org/10.6084/m9.figshare.28852529.v1.
