# Supplementary material for: Strong genetic differentiation and low genetic diversity in a habitat‐forming fucoid seaweed (Cystophora racemosa) across 850 km of its range
Source: J Phycol. 2025 May 3;61(3):539–57. doi: 10.1111/jpy.70023 (PMC12168110; doi:10.1111/jpy.70023)
Supplement: Supplementary file 1 — Figure S1. Histograms of test significance values (p‐values) for LFMM, using the adjusted genomic inflation factor of 0.9 (GIF) for SST max (°C). Figure S2. Redundancy analysis (RDA) for the combined dataset (4741 SNPs). Figure S3. Putative adaptive loci identified by four methods: BayeSCAN, PCadapt, LFMM and RDA. Figure S4. Missingness per SNP (%) for the neutral (n = 4217 SNPs) and adaptive (n = 70 SNPs) datasets for all six sites combined. Figure S5. Allele frequencies of adaptive loci identified by RDA and LFMM (n = 14 SNPs) for six populations, whereby data is not imputed. Figure S6. Allele frequencies of adaptive loci identified by RDA and LFMM (n = 14 SNPs) for six populations, whereby data is imputed by sNMF. Figure S7. Principal Coordinates Analysis (PCoA) of the combined dataset (n = 76 individuals, n = 6 sites, 4741 SNPs). Table S1. The effect of SNP filtering cut‐offs on the number of individuals (Ind. Count), number of SNP loci (SNP count), missingness (%) per SNP, mean read depth, mean (± SD) HE, mean (±SD) F IS, overall (± SD) F ST. Table S2. Number of individuals per site (Ind. Count) before (n = 17, 239 SNPs) and after (4741 SNPs) filtering, respective site level mean missing rate on individuals (%) calculated before and after filtering. Table S3. Environmental variables values for six sites, extracted from Bio‐ORACLE version 2.0 (Assis et al., 2018). Table S4. Pearson's correlation coefficient values for pairs of environmental variables extracted from Bio‐ORACLE version 2.0 (Assis et al., 2018). Table S5. Mean missingness of adaptive loci (n = 70 SNPs) and neutral loci (n = 4421 SNPs) for six sites. Table S6. Pairwise oceanographic distance (km) between sites, calculated in melfuR. [file JPY-61-539-s001.docx]

**Supplemental Information for:**

**Strong genetic differentiation and low genetic diversity in a habitat-forming fucoid seaweed (*Cystophora racemosa*) across 850 km of its range.**

Edgeloe J.M., Samuel S., Pessarrodona A., Coleman M.A. , Batley J., Wernberg T., Wood G.V.

**Table of Contents:**

| **Figure S1:** Histograms of test significance values (p-values) for LFMM, using the adjusted genomic inflation factor of 0.9 (GIF) for SST max (°C). | Page 2 |
| --- | --- |
| **Figure S2:** Redundancy analysis (RDA) for the combined dataset (4,741 SNPs). The coloured circles represent individuals (n = 76) from six sites. The grey circles represent neutral SNPs (n = 4,421 SNPs) and red circles represent outlier SNPs (n = 320). Outlier SNPs were identified based on a loading of greater than ± 3.0 (two-tailed p-value = 0.0027). The RDA axis was significant (F_1, 75_ =4.28, adj R^2^ = 0.04, p < 0.01). | Page 3 |
| **Figure S3:** Putative adaptive loci identified by four methods: BayeSCAN, PCadapt, LFMM and RDA. Loci found in at least two methods were included in the adaptive dataset (n = 70 SNPs). | Page 4 |
| **Figure S4:** Missingness per SNP (%) for the neutral (n= 4,217 SNPs) and adaptive (n = 70 SNPs) datasets for all six sites combined. Adaptive loci are highlighted in “red” and neutral loci are highlighted in “grey”. | Page 5 |
| **Figure S5:** Allele frequencies of adaptive loci identified by RDA and LFMM (n = 14 SNPs) for six populations, whereby data is not imputed. Black points correspond to individual’s allele frequencies (n = 76 individuals total across all six sites), with red points corresponding to missing data. | Page 6 |
| **Figure S6:** Allele frequencies of adaptive loci identified by RDA and LFMM (n = 14 SNPs) for six populations, whereby data is imputed by sNMF. Black points correspond to individual’s allele frequencies (n = 76 individuals total across all six sites). | Page 7 |
| **Figure S7:** Principal Coordinates Analysis (PCoA) of the combined dataset (n = 76 individuals, n = 6 sites, 4,741 SNPs). Different colours represent different sites, with points corresponding to individuals | Page 8 |
| **Table S1:** The effect of SNP filtering cut-offs on the number of individuals (Ind. Count), number of SNP loci (SNP count), missingness (%) per SNP, mean read depth, mean (± SD) H_E,_ mean (± SD) F_IS_, overall (± SD) F_ST_. Quality filtering adjusted for max-missing (%) data allowed per SNP, the minor allele frequency (MAF), minimum read depth per locus across all individuals (MinDP (X)) and reproducibility.  Highlighted rows are filtering parameters used in the next step of filtering. | Page 9 |
| **Table S2:** Number of individuals per site (Ind. Count) before (n= 17, 239 SNPs) and after (4,741 SNPs) filtering, respective site level mean missing rate on individuals (%) calculated before and after filtering. Note, mean missing rate after filtering not calculated for ‘Canal Rocks’ site due to exclusion of site from final dataset. | Page 10 |
| **Table S3:** Environmental variables values for six sites, extracted from Bio-ORACLE version 2.0 (Assis *et al.* 2018). | Page 10 |
| **Table S4:** Pearson’s correlation coefficient values for pairs of environmental variables extracted from Bio-ORACLE version 2.0 (Assis *et al.* 2018). | Page 11 |
| **Table S5:** Mean missingness of adaptive loci (n = 70 SNPs) and neutral loci (n 4,421 SNPs) for six sites. | Page 12 |
| **Table S6:** Pairwise oceanographic distance (km) between sites, calculated in *melfuR.* | Page 12 |


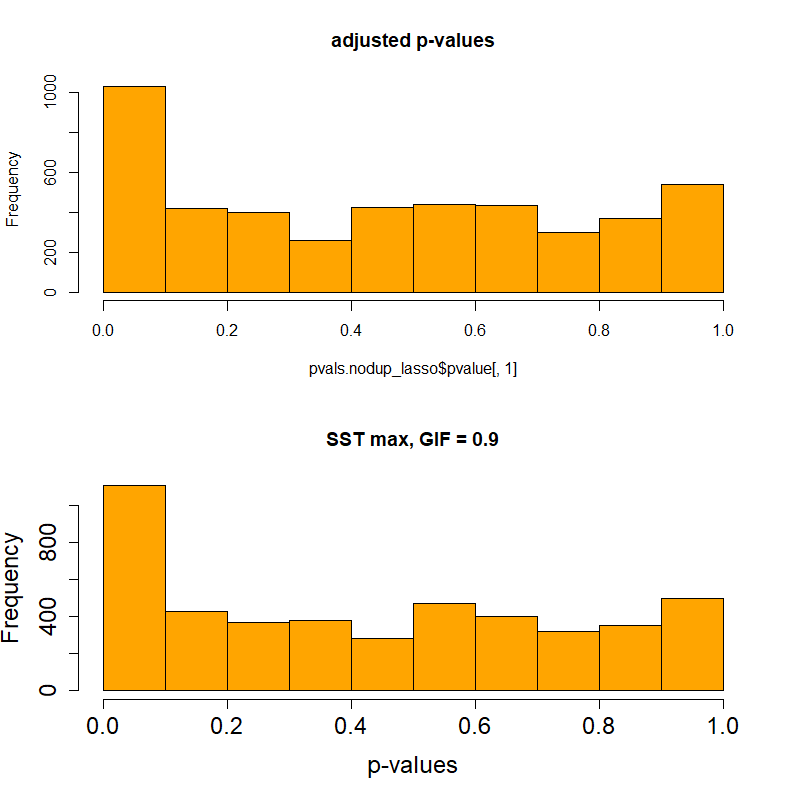


**Figure S1:** Histograms of test significance values (*p*-values) for LFMM, using the adjusted genomic inflation factor of 0.9 (GIF) for SST max (°C).


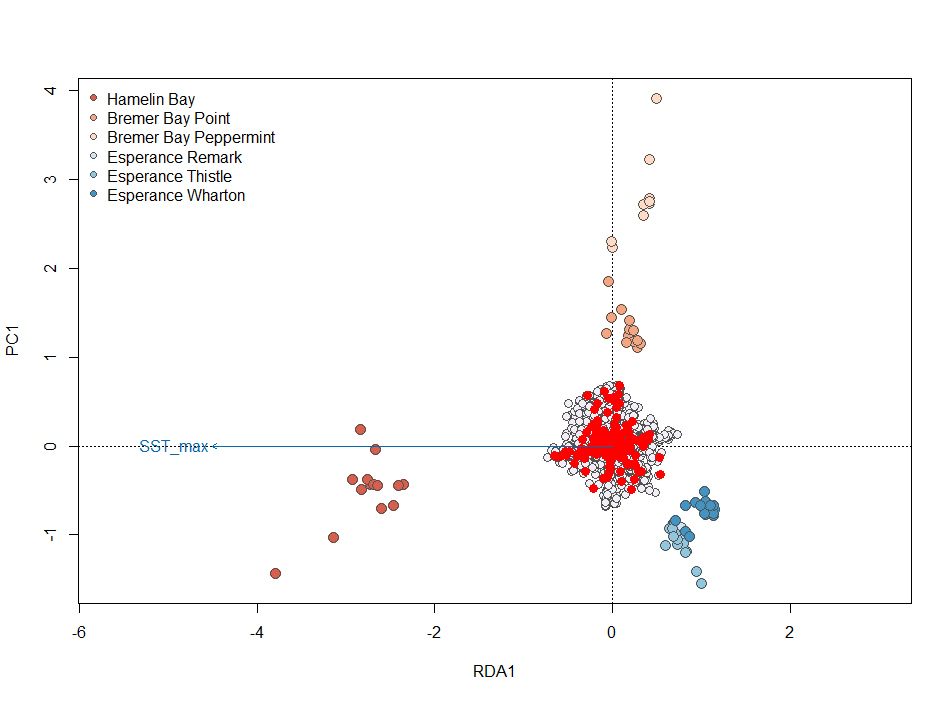


**Figure S2:** Redundancy analysis (RDA) for the combined dataset (4,741 SNPs). The coloured circles represent individuals (*n* = 76) from six sites. The grey circles represent neutral SNPs (*n* = 4,421 SNPs) and red circles represent outlier SNPs (n = 320). Outlier SNPs were identified based on a loading of greater than ± 3.0 (two-tailed *p*-value = 0.0027). The RDA axis was significant (*F*_1, 75_ =4.28, adj *R*^2^ = 0.04, *p* < 0.01)


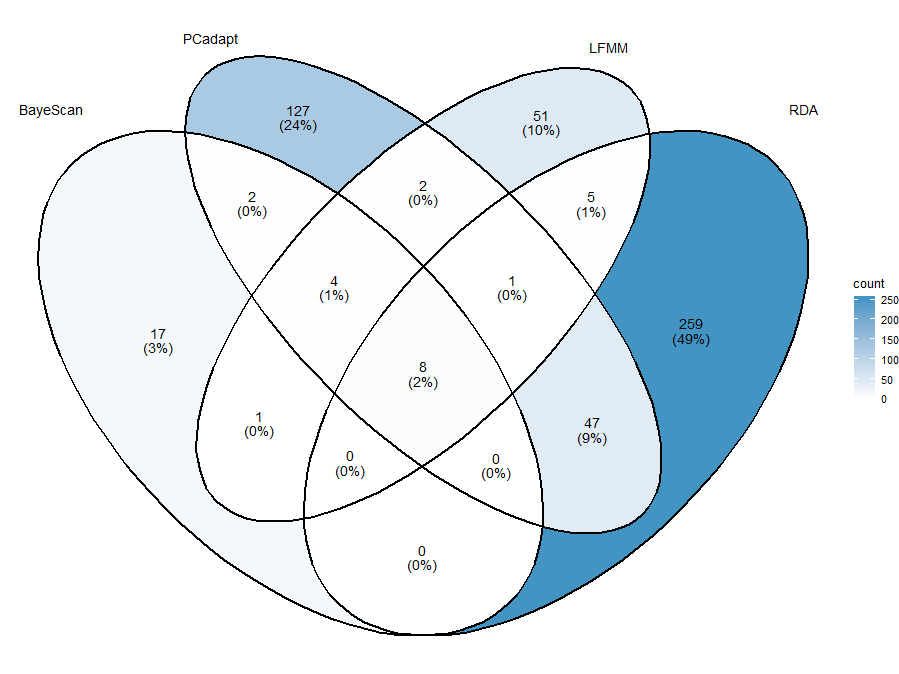


**Figure S3:** Putative adaptive loci identified by four methods: BayeSCAN, PCadapt, LFMM and RDA. Loci found in at least two methods were included in the adaptive dataset (*n* = 70 SNPs).


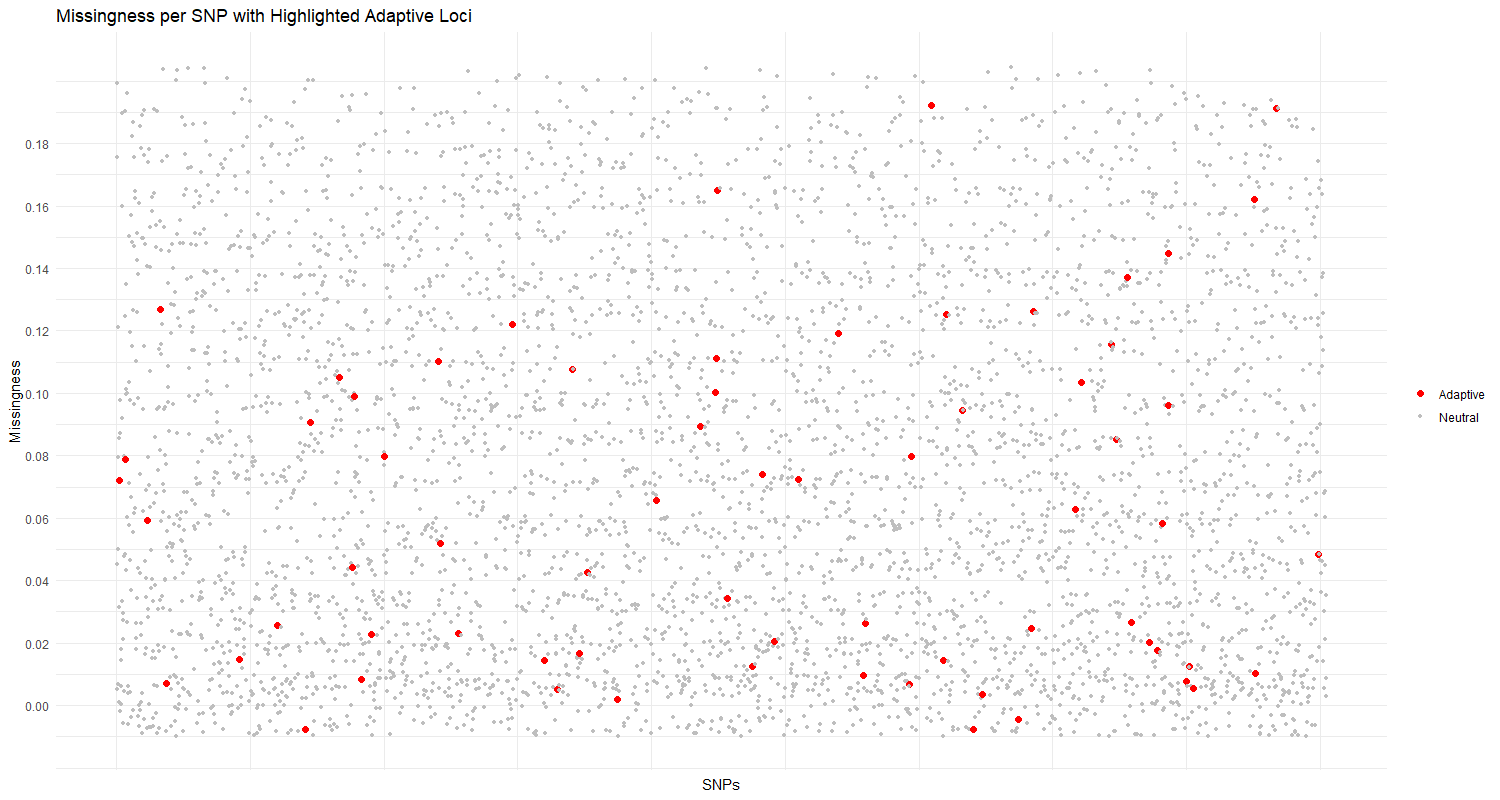


**Figure S4:** Missingness per SNP (%) for the neutral (*n =* 4,217 SNPs) and adaptive (*n* = 70 SNPs) datasets for all six sites combined. Adaptive loci are highlighted in “red” and neutral loci are highlighted in “gray.”


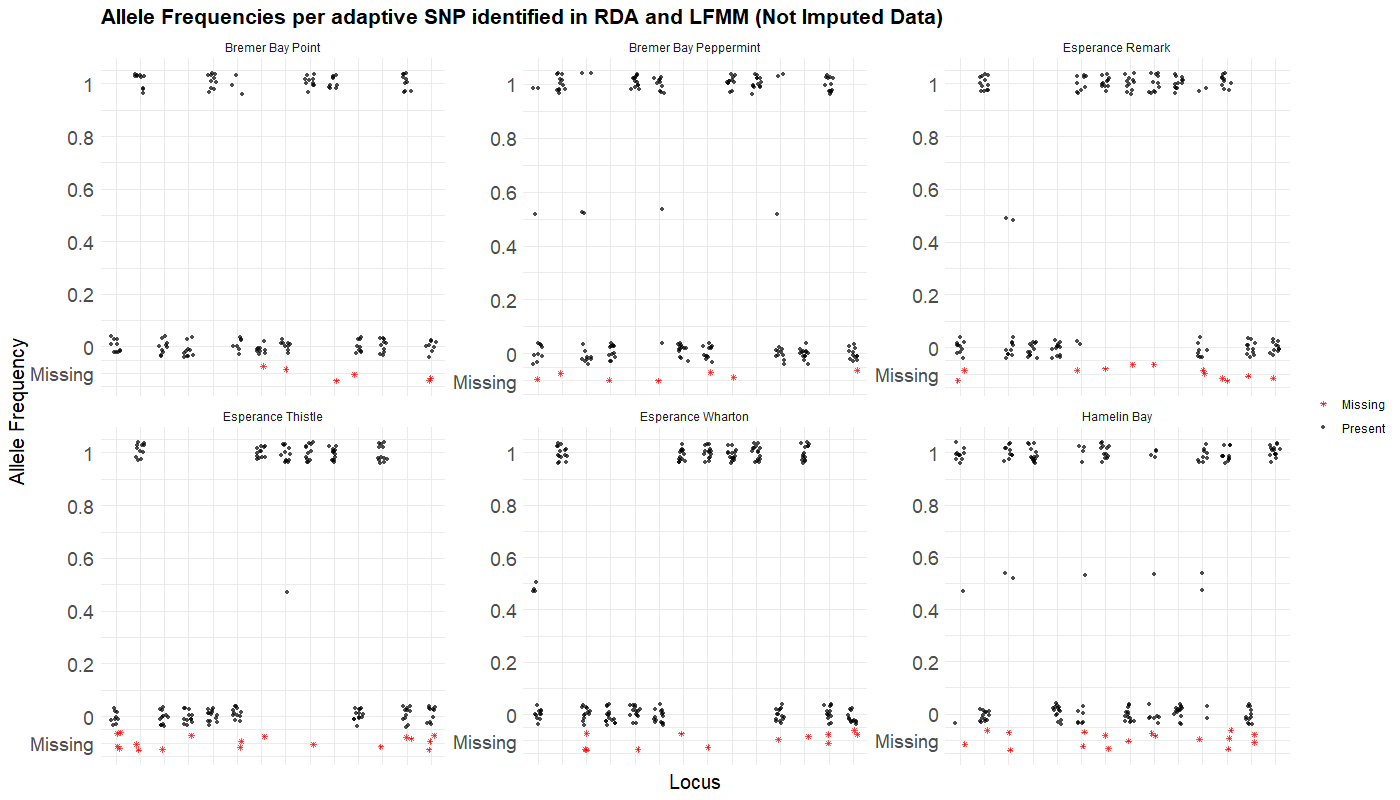


**Figure S5:** Allele frequencies of adaptive loci identified by RDA and LFMM (*n* = 14 SNPs) for six populations, whereby data is not imputed. Black points correspond to individual’s allele frequencies (*n* = 76 individuals total across all six sites), with red points corresponding to missing data.


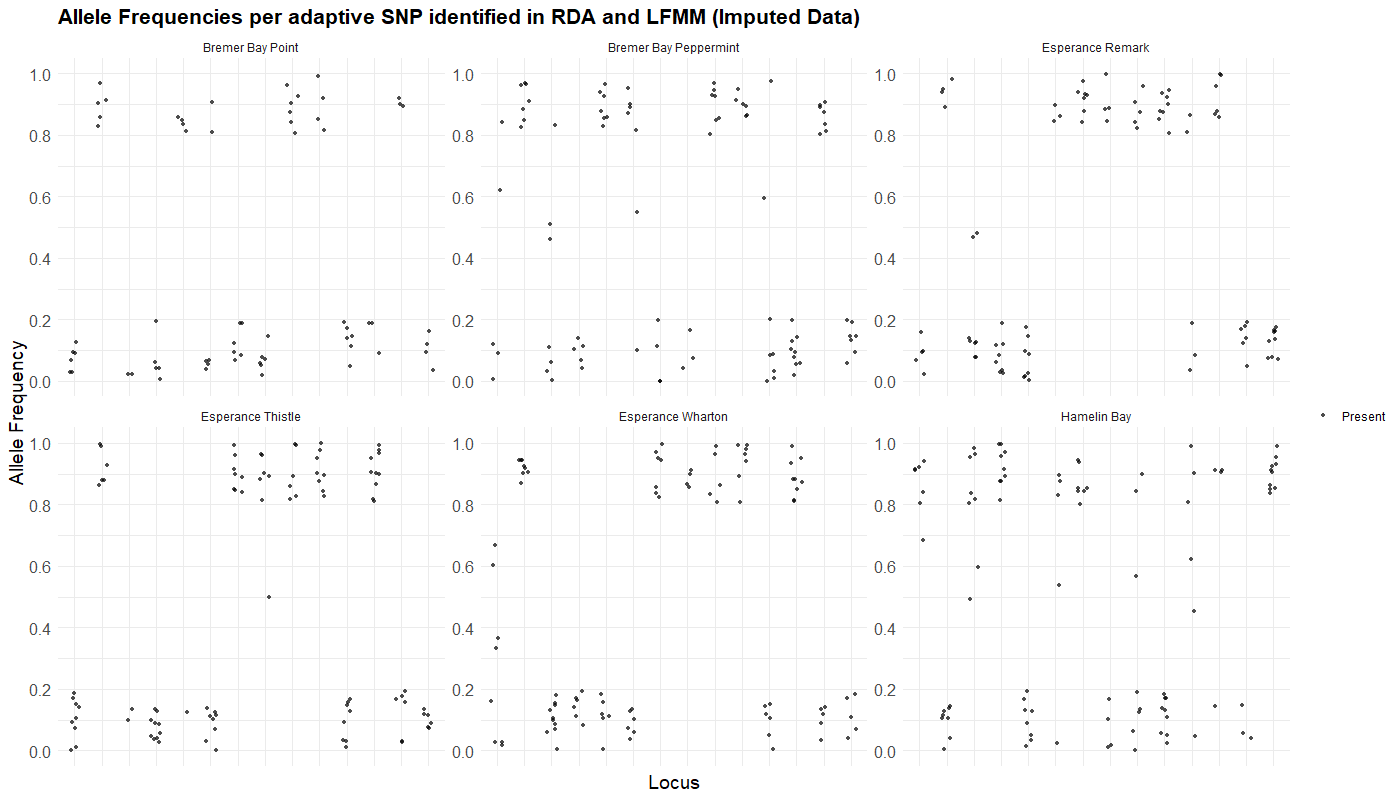


**Figure S6:** Allele frequencies of adaptive loci identified by RDA and LFMM (*n* = 14 SNPs) for six populations, whereby data is imputed by sNMF. Black points correspond to individual’s allele frequencies (*n* = 76 individuals total across all six sites).


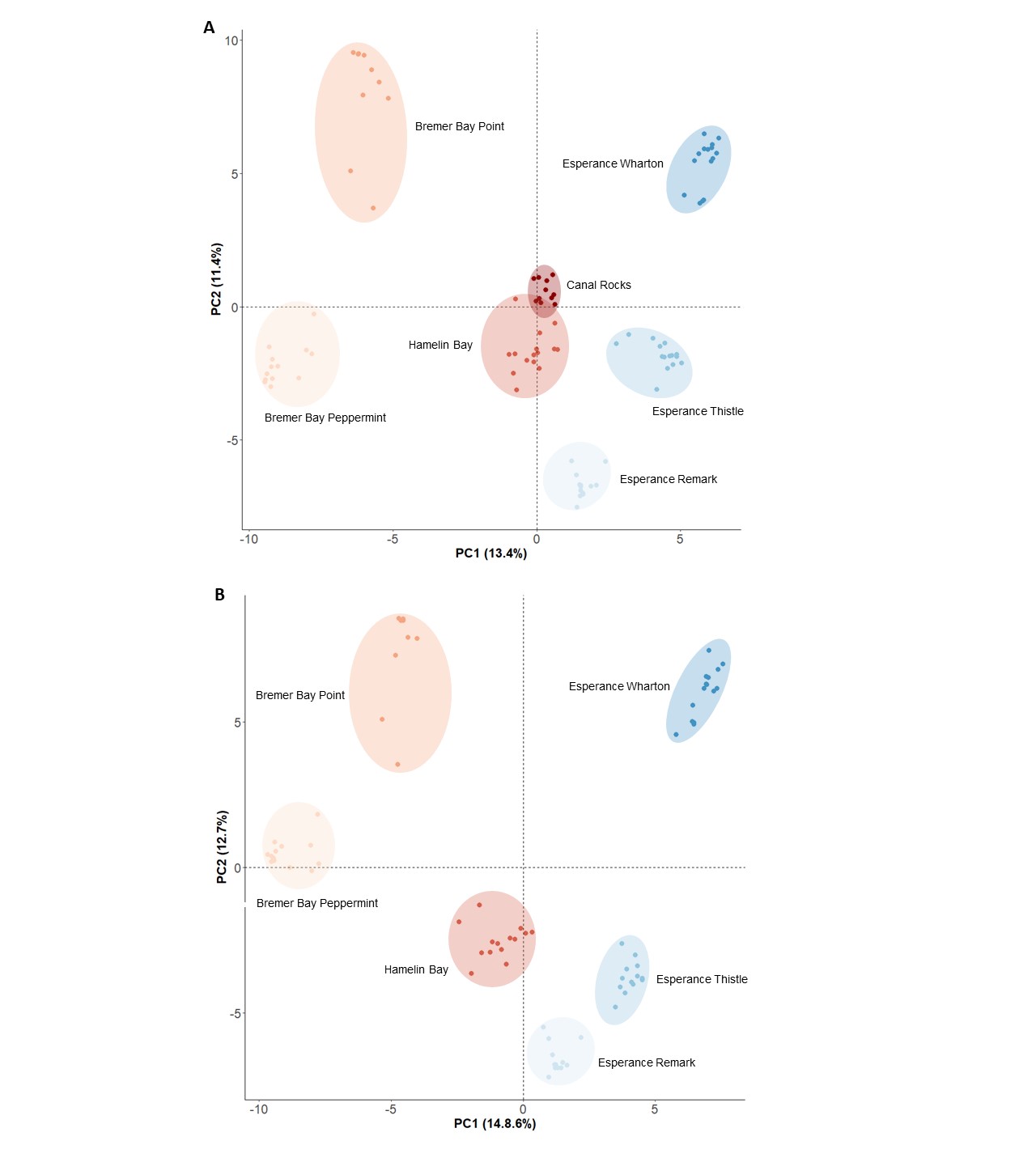


**Figure S7:** Principal Coordinates Analysis (PCoA) of the combined dataset (*n* = 76 individuals, *n* = 6 sites, 4741 SNPs). Different colours represent different sites, with points corresponding to individuals

**Table S1:** The effect of SNP filtering cut-offs on the number of individuals (Ind. Count), number of SNP loci (SNP count), missingness (%) per SNP, mean read depth, mean (± *SD*) *H*_E,_ mean (± *SD*) *F*_IS_, overall (± *SD*) *F*_ST_. Quality filtering adjusted for max-missing (%) data allowed per SNP, the minor allele frequency (MAF), minimum read depth per locus across all individuals (MinDP (X)) and reproducibility.  Highlighted rows are filtering parameters used in the next step of filtering.

| **Quality filter** | **cut-off** | **Ind. Count** | **SNP count** | **Missingness %** | **Mean Read Depth** | **Mean (± *SD*) *H*_E_** | **Mean (± *SD*) *F*_IS_** | **Overall (± *SD*) *F*_ST_** |
| --- | --- | --- | --- | --- | --- | --- | --- | --- |
| Reference dataset |  | 98 | 17239 | 23.78 | 4.52 | 0.05 (0.02) | 0.58 (0.08) | 0.49 (0.08) |
| Max-missing (%) | <30 | 76 | 14999 | 10.26 | 4.24 | 0.04 (0.02) | 0.43 (0.19) | 0.51 (0.11) |
|  | <20 | 76 | 12784 | 7.80 | 4.32 | 0.04 (0.02) | 0.39 (0.17) | 0.50 (0.11) |
|  | <10 | 76 | 8036 | 4.03 | 5.01 | 0.04 (0.02) | 0.34 (0.15) | 0.50 (0.11) |
|  | <5 | 76 | 4568 | 1.83 | 6.30 | 0.05 (0.02) | 0.29 (0.14) | 0.50 (0.11) |
|  | 0 | 76 | 1214 | 0.00 | 8.99 | 0.06 (0.02) | 0.17 (0.11) | 0.49 (0.10) |
| MAF | 0.01 | 76 | 5528 | 7.66 | 7.51 | 0.08 (0.04) | 0.44 (0.19) | 0.52 (0.11) |
|  | 0.02 | 76 | 3771 | 6.99 | 9.5 | 0.11 (0.05) | 0.42 (0.18) | 0.53 (0.11) |
|  | 0.05 | 76 | 2815 | 6.82 | 10.72 | 0.12 (0.06) | 0.41 (0.18) | 0.54 (0.11) |
| MinDP (X) cut off for fixed MAF (0.01) | >2 | 76 | 5362 | 7.66 | 7.5 | 0.08 (0.04) | 0.44 (0.19) | 0.52 (0.11) |
|  | >3 | 76 | 2768 | 7.62 | 7.67 | 0.08 (0.04) | 0.43 (0.19) | 0.53 (0.11) |
|  | >4 | 76 | 1651 | 7.77 | 7.83 | 0.08 (0.04) | 0.43 (0.19) | 0.53 (0.11) |
|  | >5 | 76 | 1143 | 7.80 | 7.5 | 0.08 (0.04) | 0.43 (0.19) | 0.52 (0.11) |
| Max-missing cutoff (individuals) for min DP (>2) | <35 | 76 | 5362 | 7.66 | 7.49 | 0.08 (0.04) | 0.44 (0.19) | 0.52 (0.11) |
|  | <30 | 76 | 5362 | 7.66 | 7.49 | 0.08 (0.04) | 0.44 (0.19) | 0.52 (0.11) |
|  | <25 | 76 | 5362 | 7.66 | 7.49 | 0.08 (0.04) | 0.44 (0.19) | 0.52 (0.11) |
|  | <20 | 73 | 5362 | 7.05 | 7.48 | 0.08 (0.04) | 0.43 (0.19) | 0.52 (0.11) |
|  | <15 | 70 | 5362 | 6.59 | 7.45 | 0.09 (0.03) | 0.51 (0.07) | 0.50 (0.08) |
|  | <10 | 58 | 5362 | 5.53 | 7.40 | 0.08 (0.03) | 0.49 (0.06) | 0.50 (0.08) |
| Reproducibility | 0.96 | 76 | 4741 | 7.76 | 7.20 | 0.07 (0.04) | 0.53 (0.23) | 0.55 (0.12) |
|  | 0.97 | 76 | 4370 | 7.84 | 7.14 | 0.07 (0.03) | 0.54 (0.24) | 0.55 (0.12) |
|  | 0.98 | 76 | 4185 | 8.08 | 6.90 | 0.07 (0.03) | 0.57 (0.25) | 0.56 (0.12) |
|  | 0.99 | 76 | 4185 | 8.09 | 6.90 | 0.07 (0.03) | 0.57 (0.25) | 0.56 (0.12) |

**Table S2:** Number of individuals per site (Ind. Count) before (*n*= 17, 239 SNPs) and after (4741 SNPs) filtering, respective site level mean missing rate on individuals (%) calculated before and after filtering. Note, mean missing rate after filtering not calculated for ‘Canal Rocks’ site due to exclusion of site from final dataset.

| **Site** | **Ind. Count (Before)** | **Ind. Count (After)** | **Mean Missing Rate (Ind)- Before Filtering** | **Mean Missing Rate (Ind)- After Filtering** |
| --- | --- | --- | --- | --- |
| Canal Rocks | 15 | 0 | 55.73% | **-** |
| Hamelin Bay | 15 | 14 | 20.69% | 11.04% |
| Bremer Bay Point | 10 | 10 | 18.06% | 8.44% |
| Bremer Bay Peppermint | 14 | 12 | 18.04% | 5.68% |
| Esperance Remark | 14 | 12 | 21.25% | 7.16% |
| Esperance Thistle | 15 | 13 | 20.48% | 8.55% |
| Esperance Wharton | 15 | 15 | 12.27% | 5.22% |

**Table S3:** Environmental variables values for six sites, extracted from Bio-ORACLE version 2.0 (Assis et al*.*, 2018)

| **Site** | **Latitude** | **Longitude** | **Sea surface temperature max (°C)** | **Current Velocity mean (m/s)** | **Nitrate mean (micro mol ⋅ m^−3^)** | **Salinity mean (PSS)** | **Light mean (E ⋅ m^−2^ ⋅ year^−1^)** |
| --- | --- | --- | --- | --- | --- | --- | --- |
| Hamelin Bay | -34.263 | 115.024 | 23.686 | 0.169 | 0.013 | 35.541 | 36.905 |
| Bremer Bay Point | -34.400 | 119.410 | 21.450 | 0.251 | 0.182 | 35.603 | 36.604 |
| Bremer Bay Peppermint | -34.400 | 119.480 | 21.508 | 0.248 | 0.178 | 35.601 | 36.708 |
| Esperance Remark | -34.045 | 121.991 | 21.09 | 0.065 | 0.012 | 35.676 | 37.642 |
| Esperance Thistle | -34.000 | 122.204 | 21.047 | 0.063 | 0.010 | 35.685 | 37.513 |
| Esperance Wharton | -33.944 | 122.559 | 20.996 | 0.063 | 0.013 | 35.686 | 37.131 |

**Table S4:** Pearson’s correlation coefficient values for pairs of environmental variables extracted from Bio-ORACLE version 2.0 (Assis et al., 2018).


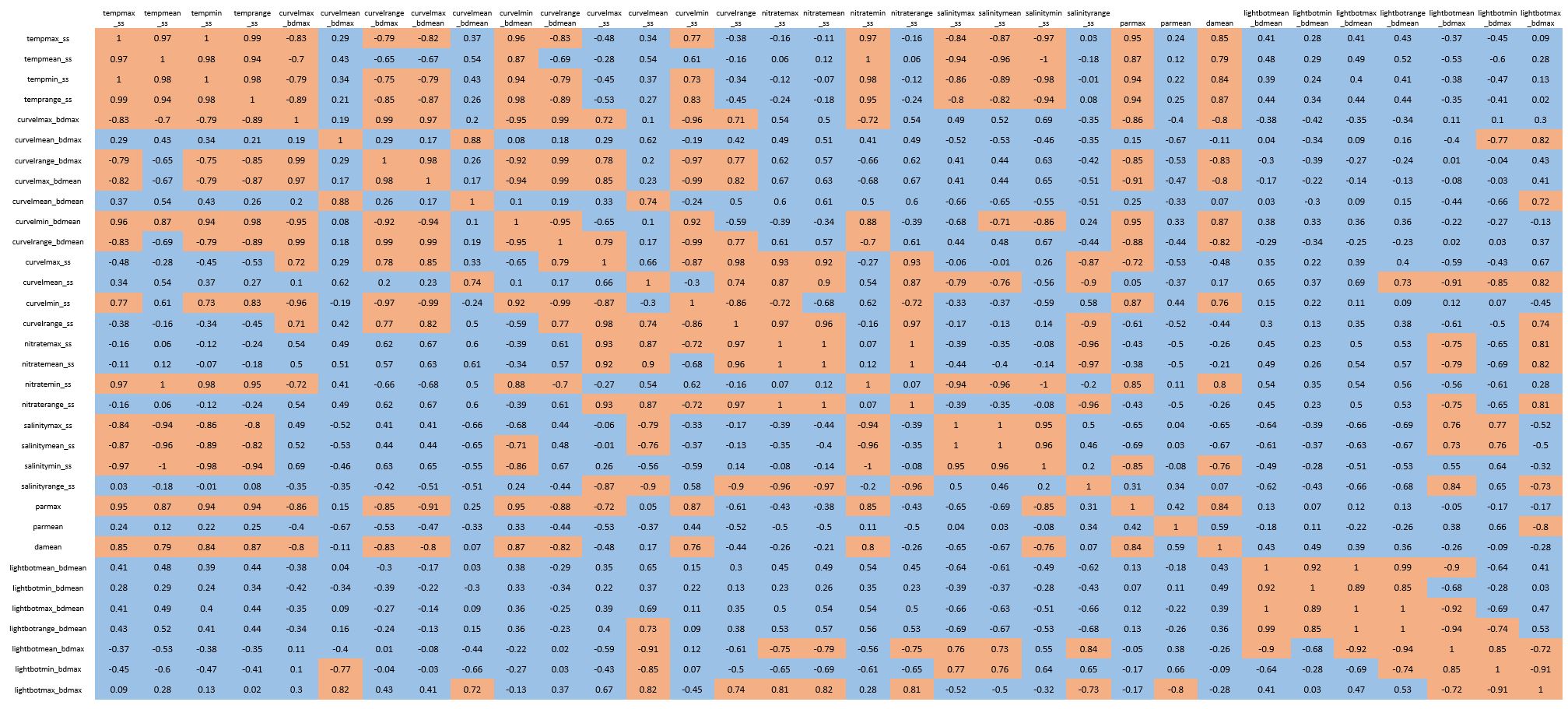


**Table S5:** Mean missingness of adaptive loci (*n* = 70 SNPs) and neutral loci (*n* = 4421 SNPs) for six sites.

| **Site** | **Mean Missingness (Adaptive, *n* = 70 SNPs)** | **Mean Missingness (Neutral, *n* = 4421 SNPs)** |
| --- | --- | --- |
| Hamelin Bay | 8.27% | 11.1% |
| Bremer Bay Point | 7.43% | 8.48% |
| Bremer Bay Peppermint | 3.19% | 5.71% |
| Esperance Remark | 5.71% | 7.21% |
| Esperance Thistle | 8.24% | 8.52% |
| Esperance Wharton | 3.71% | 5.20% |

**Table S6:** Pairwise oceanographic distance (km) between sites, calculated in melfuR*.*

| **Site** | Hamelin Bay | Bremer Bay Point | Bremer Bay Peppermint | Esperance Remark | Esperance Thistle |
| --- | --- | --- | --- | --- | --- |
| Bremer Bay Point | 466.206 |  |  |  |  |
| Bremer Bay Peppermint | 468.735 | 6.698 |  |  |  |
| Esperance Remark | 709.795 | 250.842 | 244.144 |  |  |
| Esperance Thistle | 732.124 | 273.303 | 266.604 | 22.525 |  |
| Esperance Wharton | 764.351 | 305.728 | 299.030 | 55.047 | 34.543 |

**References supplementary material.**

Assis, J., Tyberghein, L., Bosch, S., Verbruggen, H., Serrão, E. A., & De Clerck, O. (2018). Bio-ORACLE v2.0: Extending marine data layers for bioclimatic modelling. *Global ecology and biogeography*, *27*(3/4), 277-284. <https://doi.org/10.1111/geb.12693>
